# *Fusobacterium nucleatum* as a two-hit amplifier in colorectal cancer pathogenesis: intersection of local and systemic mechanisms via the gut–kidney axis

**DOI:** 10.20407/fmj.2025-042

**Published:** 2026-02-28

**Authors:** Hidehisa Shimizu

**Affiliations:** 1 Faculty of Life and Environmental Sciences, Shimane University, Matsue, Shimane, Japan; 2 Graduate School of Natural Science and Technology, Shimane University, Matsue, Shimane, Japan; 3 Estuary Research Center, Shimane University, Matsue, Shimane, Japan; 4 Interdisciplinary Center for Science Research, Shimane University, Matsue, Shimane, Japan; 5 The United Graduate School of Agricultural Sciences, Tottori University, Tottori, Tottori, Japan; 6 Institute of Agricultural and Life Sciences, Academic Assembly, Shimane University, Matsue, Shimane, Japan

**Keywords:** Colorectal cancer, *Fusobacterium nucleatum*, Gut microbiota, Gut–kidney axis, Uremic toxin

## Abstract

Colorectal cancer (CRC) is a complex malignancy driven by genetic, environmental, and lifestyle factors. A critical contributor to CRC tumor initiation and progression is gut microbial dysbiosis. *Fusobacterium nucleatum*, an oral commensal bacterium, is a potent promoter of malignancy rather than a passive colonizer. This review examines the molecular mechanisms by which *F. nucleatum* promotes CRC progression, focusing on one indirect systemic and three direct local mechanisms. First, the lipopolysaccharide–Toll-like receptor 4 axis sustains a chronic inflammatory loop that remodels the tumor microenvironment. Second, the fibroblast activation protein 2–T cell immunoglobulin and immunoreceptor tyrosine-based inhibitory motif domain interaction facilitates immune evasion by suppressing T cell and natural killer cell cytotoxic activity. Third, the *Fusobacterium* adhesin A–E-cadherin/Wnt pathway acts as a potent “second hit,” amplifying β-catenin signaling and driving proliferation in cells with pre-existing mutations in adenomatous polyposis coli. Beyond these local effects, this review highlights a novel indirect mechanism involving the gut–kidney axis. Specifically, *F. nucleatum* is hypothesized to contribute to systemic levels of indoxyl sulfate, a uremic toxin that reinforces a systemic feedback loop via pro-proliferative signaling. These synergistic mechanisms collectively remodel the tumor microenvironment by promoting immune suppression, chronic inflammation, and metabolic reprogramming. Within this framework, *F. nucleatum* collaborates with genetic lesions to accelerate disease progression. This framework provides a crucial foundation for developing innovative diagnostics and microbe-targeted therapies—such as phage therapy and vaccines—to substantially improve the prognosis of patients with CRC.

## Introduction

The human gastrointestinal tract is home to a complex microbial ecosystem, the gut microbiota, which is vital for host physiology.^1,2^ Dysbiosis, an imbalance in the gut microbiota, has been increasingly implicated in various pathologies, including colorectal cancer (CRC).^3–8^ Worldwide, CRC has a high incidence and significant morbidity and mortality. The role of the gut microbiota in its pathogenesis has increasingly become a focus of intensive research. The gut microbial community is not static as CRC progresses; it rather undergoes dynamic, stage-specific changes in composition and metabolic function (Table 1).^6^ Among the various microorganisms involved, *Fusobacterium nucleatum* (*F. nucleatum*) has emerged as a keystone pathogen in CRC pathogenesis.^7^ This gram-negative anaerobic bacterium is typically an oral commensal and is rarely detected in the lower gastrointestinal tract of healthy individuals.^9^ However, its abundance is significantly higher in the tumor tissue and fecal samples of CRC patients than in normal tissue. The abundance of *F. nucleatum* also correlates with tumor stage and malignancy and is associated with poor prognosis and chemotherapy resistance.^10–12^ For instance, a meta-analysis revealed that a high abundance of *F. nucleatum* in tumor tissue is significantly associated with poorer overall survival (hazard ratio: 1.87; 95% confidence interval: 1.12–3.11).^13^

This review is based on a literature search of PubMed and Google Scholar for articles published up to August 21, 2025, using keywords such as “*Fusobacterium nucleatum*,” “colorectal cancer,” “gut–kidney axis,” and “indoxyl sulfate.” The search prioritized primary research articles detailing molecular mechanisms, key epidemiological studies, and recent reviews that collectively provide a basis for the synergistic model presented in this review. While previous reviews have detailed the individual pathogenic mechanisms of *F. nucleatum*, a comprehensive model integrating these pathways remains underdeveloped. This review aims to advance a central hypothesis: *F. nucleatum* is not merely an opportunistic passenger but is a potent promoter that actively and synergistically drives CRC progression through a multi-layered attack. In this context, a promoter is defined as a microbe that does not initiate the primary genetic mutations (the “first hit”) but rather exploits an existing pre-malignant or malignant state to accelerate tumor growth, enhance immune evasion, and create a pro-inflammatory microenvironment.

A unique aspect of this study is the incorporation of the gut–kidney axis, a perspective often overlooked in CRC-focused reviews. By examining the interplay between CRC and chronic kidney disease (CKD), this review proposes a more holistic view in which *F. nucleatum* acts not only as a local tumor promoter but also as a key player in systemic multi-organ pathology. To delineate this multi-layered attack, we first detail the direct mechanisms of *F. nucleatum* within the tumor microenvironment, and then explore its indirect systemic effects that operate via the gut–kidney axis.

## Phylogenetic context of *F. nucleatum* and its translocation to the colorectum

*F. nucleatum* is a primary commensal of the oral cavity known for its role in periodontal disease; however, its presence within the colorectal tumor microenvironment represents a fascinating and critical aspect of colorectal cancer pathology.^14^ Translocation is hypothesized to occur via two main routes: direct passage through the gastrointestinal tract and hematogenous dissemination. While the oral–gastrointestinal route is plausible, evidence indicates that hematogenous spread of bacteria from the oral cavity may be a more effective mechanism. Chronic periodontal disease, often associated with transient bacteremia, provides a potential portal for *F. nucleatum* to enter the bloodstream.^15^ This hypothesis is supported by epidemiological studies linking poor oral hygiene to a higher risk of CRC.^16^ Furthermore, animal models have demonstrated that intravenous injection of *F. nucleatum* leads to more effective colonization of CRC tumors than oral gavage, indicating that the hematogenous route is a key mechanism for its distant dissemination.^17^

Intriguingly, recent studies have revealed significant phylogenetic diversity within *F. nucleatum*, with different subspecies and strains exhibiting varying degrees of virulence. For instance, the *Fusobacterium nucleatum* subsp. *animalis* (Fna) C2 clade is consistently more prevalent and abundant in CRC tissues than other strains, indicating that a specific and highly pathogenic subpopulation drives pro-tumorigenic effects.^18^ Recent phylogenetic analyses have begun to elucidate the roles of specific *F. nucleatum* subpopulations in CRC progression. A particularly groundbreaking 2024 study by Zepeda-Rivera et al. proposed that Fna, previously considered a single subspecies, actually diverges into two distinct clades: Fna C1 and Fna C2. Their comprehensive genomic analysis demonstrated that the Fna C2 clade predominantly occupies the CRC tumor niche. To elucidate the genetic basis for the Fna C2 clade’s adaptation to the CRC microenvironment, Zepeda-Rivera et al. performed comparative genomics against Fna C1. This analysis identified 195 Fna C2-associated genetic factors that likely provide enhanced capabilities for gastrointestinal colonization and metabolism. Specifically, Fna C2 demonstrated increased resistance to gastric acid exposure and a superior capacity for using nutrients that are abundant in the gastrointestinal tract, such as ethanolamine and 1,2-propanediol. These characteristics are thought to enhance the ability of Fna C2 to survive transit from the oral cavity and to allow efficient colonization and proliferation within the tumor microenvironment. Indeed, CRC model mice administered Fna C2 exhibited a greater number of intestinal adenomas compared with those administered Fna C1. Furthermore, their gut metabolome shifted to promote oxidative stress and inflammatory pathways, corroborating the unique capacity of Fna C2 to drive tumor progression.^19^ However, it is crucial to note the rapid evolution of this field. The classification proposed by Zepeda-Rivera et al. has since been contested. Multiple research groups have argued that the clade designated Fna C1 was, in fact, a misidentification of the previously described species, *Fusobacterium watanabei*. This taxonomic dispute reframes the comparison from one between two intraspecific clades to one between two distinct species. Consequently, the identified genetic differences may represent simple inter-species variation rather than specific adaptations to the CRC microenvironment. Nonetheless, the central observation remains robust: the Fna C2 lineage is the primary pathogenic actor found within the CRC microenvironment. The superior colonization and proliferation capabilities of Fna C2 provide a foundation upon which the other pathogenic mechanisms discussed herein are executed. In conclusion, the specificity of the Fna C2 clade likely stems from its enhanced capacity for survival and colonization within the gastrointestinal tract. This superior fitness allows Fna C2 to efficiently reach the tumor microenvironment. Once there, it deploys the potent immunosuppressive and oncogenic mechanisms common to the *F. nucleatum* species, thereby functioning as a key driver of CRC progression.

## Direct carcinogenic mechanisms of *F. nucleatum*

### Chronic inflammation: the LPS/TLR4/NFκB pathway

One of the most prominent mechanisms by which *F. nucleatum* contributes to CRC pathogenesis is by manipulating the host’s inflammatory response.^20,21^ The *F. nucleatum* cell wall lipopolysaccharide (LPS) activates Toll-like receptor 4 (TLR4) in host cells. As a key receptor in the innate immune response, TLR4 activation initiates a crucial intracellular signaling cascade. Upon binding of *F. nucleatum* LPS to TLR4 on CRC cells, signaling is triggered via myeloid differentiation primary response gene 88 (MyD88), which leads to activation of the transcription factor, nuclear factor kappa B (NFκB).^21^ NFκB is a key regulator of genes involved in cell proliferation, survival, and inflammation, and its activation results in the robust production of pro-inflammatory cytokines, including interleukin (IL)1β, IL6, IL8, tumor necrosis factor alpha (TNFα), monocyte chemoattractant protein 1 (MCP1), and IL17A.^21,22^ This inflammatory mechanism is further complicated by the involvement of additional pathways. *F. nucleatum* can also activate NFκB via a MyD88-independent pathway, specifically through the alpha-kinase 1/TNF receptor-associated factor-interacting protein with forkhead-associated domain/TNF receptor-associated factor 6 axis, which is triggered by the bacterial metabolite, ADP-glycero-β-_D_-manno-heptose.^20^ Furthermore, *F. nucleatum* upregulates microRNA-21 (miR-21) expression via the TLR4 pathway. miR-21 subsequently suppresses various tumor suppressor target genes, such as the RAS GTPase-activating protein, RAS p21 protein activator 1, thereby amplifying NFκB activity.^21^ These mechanisms create a positive feedback loop, wherein inflammation is not only initiated but also sustained and amplified, establishing a chronic and self-maintaining inflammatory loop that actively remodels the tumor microenvironment into a state favorable to cancer cells. This pathway is summarized in Table 2.

### A multi-pronged strategy for immune evasion

For CRC to progress, tumor cells must evade immune surveillance, a process that *F. nucleatum* actively facilitates through multiple mechanisms. The most direct mechanism involves its outer membrane adhesin, fibroblast activation protein 2 (Fap2), which directly engages with the inhibitory receptor, T cell immunoglobulin and immunoreceptor tyrosine-based inhibitory motif domain (TIGIT), on natural killer (NK) cells and cytotoxic CD8^+^ T cells.^23^ The Fap2–TIGIT interaction is a critical mediator of immune evasion as it suppresses the cytotoxic activity of these crucial antitumor effectors, thereby protecting tumor cells from destruction.^23^ Beyond this direct engagement, *F. nucleatum* orchestrates a broadly immunosuppressive tumor microenvironment. The chronic inflammation induced via the LPS/TLR4 pathway plays a critical role in reshaping the immune landscape. This inflammatory milieu is rich in cytokines, such as IL6 and TNFα, which promote the polarization of tumor-associated macrophages toward a pro-tumor, immunosuppressive M2 phenotype.^24^ These M2 macrophages not only fail to attack cancer cells but also actively suppress the function of other immune cells, including T cells, further fortifying the immune-privileged niche.^25^ The combined effect of direct TIGIT engagement and the chronic inflammatory environment leads to a profound state of T cell exhaustion, particularly affecting CD8^+^ T cells. This exhausted state is characterized by the sustained expression of multiple inhibitory receptors; as well as TIGIT, these include other key immune checkpoints such as programmed death 1, and T cell immunoglobulin and mucin-domain-containing protein 3.^26^ While *F. nucleatum* may not bind these other receptors directly, its activity creates a microenvironment that drives their upregulation on T cells, rendering them dysfunctional and unresponsive to tumor antigens. This multifaceted immune suppression explains why the presence of *F. nucleatum* is often associated with resistance to certain immunotherapies and highlights the need for combination strategies that target both the bacterium and multiple immune checkpoints to effectively restore antitumor immunity.

### Direct tumor proliferation: the FadA/Wnt pathway

In addition to its indirect pro-tumorigenic effects, *F. nucleatum* directly stimulates CRC cell proliferation via its adhesin, *Fusobacterium* adhesin A (FadA). FadA specifically binds to E-cadherin, a crucial protein for maintaining cell–cell adhesion among intestinal epithelial cells. This binding is a key mechanism for the interaction of *F. nucleatum* with specific host cells and subsequent colonization. In normal cells, E-cadherin forms a complex with β-catenin, anchoring it to the cell membrane and thereby suppressing its role in cell proliferation signaling pathways. However, FadA binding to E-cadherin disrupts this complex, leading to cytoplasmic accumulation of β-catenin. Accumulated β-catenin subsequently translocates to the nucleus, where it functions as a transcription factor to activate the Wnt signaling pathway.^27^ Recent findings have also demonstrated that FadA binding increases intracellular calcium concentration, which in turn promotes the formation of a complex between E-cadherin and the transcription factor, Krüppel-like factor 4 (KLF4). This leads to KLF4 phosphorylation and nuclear translocation, which induces the transcription of the integrin alpha 5 (*ITGA5*) gene, ultimately promoting CRC growth and metastasis.^28^

## Further refinement of the “two-hit” model: the synergy between genetic mutations and *F. nucleatum*

CRC progression is a complex process driven by the combined actions of genetic and microbial factors. This review expands Fearon and Vogelstein’s multi-hit model by positioning *F. nucleatum* as a potent second hit that dramatically accelerates disease progression.

One of the earliest genetic events in CRC development is inactivation of the tumor suppressor gene, adenomatous polyposis coli (*APC*). *APC* normally functions as a key negative regulator of the Wnt signaling pathway, which controls cell proliferation. In healthy cells, *APC* forms a complex with β-catenin, tagging it for degradation to maintain low β-catenin levels and suppression of the Wnt signal. However, a mutation in *APC* (the first hit) can reduce or abolish *APC* function, causing degradation of β-catenin to fail. Consequently, β-catenin accumulates in the cytoplasm and translocates to the nucleus, where it functions as a transcription factor that constitutively activates the Wnt signaling pathway.^29^

Following *APC* mutation, infection with *F. nucleatum* acts as a second hit. FadA binds specifically to E-cadherin on the surface of CRC cells, which not only disrupts cell-to-cell adhesion but also releases β-catenin from the cell membrane.^27^ Notably, FadA does not directly inhibit β-catenin degradation; rather, it amplifies the already dysregulated Wnt signal by promoting further β-catenin accumulation and nuclear translocation in cells that have received the first hit.

This synergistic effect between *APC* mutation and *F. nucleatum* infection dramatically accelerates the proliferation and malignancy of cancer cells, providing a clear molecular basis for the hypothesis that *F. nucleatum* is a potent promoter that works in concert with genetic mutations to accelerate tumor progression. The pathway is summarized in Table 2.

## Hypothesized systemic effects via the gut–kidney axis: the role of indoxyl sulfate

In addition to its direct local effects on tumors, it is hypothesized that *F. nucleatum* may contribute to CRC exacerbation through an indirect systemic pathway involving the host metabolism. Diet plays a key role in this process because dietary protein, the primary source of tryptophan, provides the substrate for microbial indole production. In a dysbiotic gut environment, *F. nucleatum* possesses the enzymatic machinery (e.g., tryptophanase) to directly produce indole from tryptophan.^30^ This indole is subsequently absorbed from the intestine into the bloodstream and transported to the liver, where it is metabolized to indoxyl sulfate.^31,32^

Indoxyl sulfate may act as a potent proliferative signal in CRC cells. A recent study confirmed that indoxyl sulfate exacerbates CRC by promoting the proliferation of CRC cells and enhancing their sensitivity to epidermal growth factor signaling via the activation of two key pathways: AKT/β-catenin/c-Myc and aryl hydrocarbon receptor (AHR)/c-Myc pathways.^33,34^ In addition to its proliferative effects, indoxyl sulfate exerts multifaceted actions on other pathological mechanisms. As a key uremic toxin, indoxyl sulfate contributes to the breakdown of the intestinal epithelial barrier, creating a “leaky gut,” which facilitates the systemic translocation of bacterial toxins such as LPS.^32,35^ This systemic inflammation, compounded by the pro-proliferative effects of indoxyl sulfate, is hypothesized to create a vicious cycle that could fuel CRC progression.

However, a critical unanswered question is the quantitative contribution of *F. nucleatum* to the total gut indole pool, particularly in comparison with other highly abundant indole-producing commensals, such as *Escherichia coli*. Validating the gut–kidney axis hypothesis hinges on demonstrating that *F. nucleatum* is not just a minor contributor but a significant driver of systemic indoxyl sulfate levels. To address this, future studies should move beyond correlation and employ a more quantitative and functional approach. A combination of metabolomic analysis to precisely measure indole and indoxyl sulfate concentrations and shotgun metagenomic sequencing to quantify the abundance of tryptophanase genes from specific bacteria will be essential. Furthermore, gnotobiotic mouse models colonized with defined bacterial communities (e.g., CRC patient-derived flora with and without *F. nucleatum* or co-colonized with *F. nucleatum* and *E. coli*) would provide the definitive experimental evidence needed to dissect its specific contribution to the systemic uremic toxin load.

## The gut–kidney axis: a bidirectional relationship

To fully understand the impact of *F. nucleatum* on both CRC and CKD, it is essential to consider the gut–kidney axis, a complex bidirectional communication pathway.^31,36–38^ The relationship between the gut microbiota and kidney disease is not unidirectional. While conventional research has primarily focused on how gut bacteria influence the host, the inverse—how kidney disease fundamentally alters the gut environment to favor the growth of pathogenic bacteria such as *F. nucleatum*—is an equally crucial aspect.

As CKD progresses, uremic toxins, such as urea, accumulate in the body. These toxins are excreted into the gut lumen, where they directly damage intestinal epithelial cells and compromise tight junctions. This process leads to increased intestinal permeability, a condition commonly referred to as leaky gut.^38,39^ Furthermore, the accumulation of uremic toxins and changes in intestinal pH and motility create selective pressure that favors the proliferation of specific bacteria. For instance, bacteria possessing urease (to convert urea into ammonia) and those that metabolize aromatic amino acids, such as tryptophan, to produce indole are specifically enriched.^38,40^ *F. nucleatum* is hypothesized to thrive in this altered environment, benefiting from the host’s pathology that provides a growth-promoting niche.

### The downstream impact of *F. nucleatum* on the kidney and CRC

The mechanism by which *F. nucleatum* contributes to renal damage is complex. Beyond its role in systemic inflammation, it is hypothesized that *F. nucleatum*-derived indole is metabolized into indoxyl sulfate, which directly induces renal tubular injury and interstitial fibrosis by promoting oxidative stress and activating inflammatory pathways (e.g., p53, NFκB, signal transducer and activator of transcription 3, and mitogen-activated protein kinases) within renal cells.^41–46^ This process, in turn, exacerbates the underlying pathology of CKD.^31^ Conversely, the pro-proliferative effect of indoxyl sulfate on CRC cells establishes a “CRC→*F. nucleatum*↑→indoxyl sulfate↑→CKD” cycle. As CRC progresses, increased serum indoxyl sulfate concentrations associated with gut dysbiosis have been reported,^47^ suggesting *F. nucleatum* growth promotion may contribute. This contributes to renal dysfunction, further compromising the gut barrier^48^ and potentially fueling the systemic spread of bacterial products that promote CRC.

### Determination of causality in human studies

Although compelling preclinical evidence from animal models supports a potential causal link, a definitive cause-and-effect relationship between *F. nucleatum* and the progression of human CKD has yet to be established. Current human studies are largely observational and correlational. Epidemiological evidence indicates that elevated serum levels of uremic toxins, including indoxyl sulfate, are associated with adverse cardiovascular and renal outcomes.^49^ While these findings add weight to the *in vitro* and animal model data, they do not prove causation.^50–53^ Therefore, it is critical to implement prospective interventional human studies, such as those involving targeted antibiotic or microbial interventions, to rigorously establish this causal relationship. The pathophysiology of this axis is summarized in Table 3.

## The question of ordinality: a preceding role for *F. nucleatum* in CKD?

The hypothesis that a uremic environment in CKD promotes *F. nucleatum* growth is compelling; however, the reverse ordinality—whether initial colonization by *F. nucleatum* contributes to the onset or progression of CKD—remains largely undetermined. Exploring this “chicken or egg” scenario is critical to fully understand the gut–kidney axis. For instance, long-term inflammation and systemic exposure to bacterial products originating from a dysbiotic state driven by *F. nucleatum* may theoretically precede and exacerbate renal injury. To definitively answer this chicken or egg question, a shift from cross-sectional observations to a more ambitious research design is required. We strongly advocate for a large-scale, prospective, longitudinal cohort study that enrolls a diverse population, from healthy individuals to patients with early and advanced stages of CKD. In such a study, participants would provide samples at regular intervals (e.g., annually) for comprehensive multi-omics analysis over several years. This would involve:

1. Microbiome analysis: Shotgun metagenomics to precisely quantify the abundance of *F. nucleatum*, identify specific clades, such as Fna C2, and assess the functional potential of the oral and gut microbiomes.

2. Metabolomic analysis: Profiling of serum and urine to track changes in key metabolites, most notably the uremic toxin, indoxyl sulfate, alongside other microbial-derived products.

3. Clinical monitoring: Continuous tracking of established renal function markers (e.g., estimated glomerular filtration rate and albuminuria) and systemic inflammation markers (e.g., high-sensitivity C-reactive protein and cytokines).

Analysis of the temporal relationships within this longitudinal dataset will enable the statistical determination of whether an increase in *F. nucleatum* abundance and indoxyl sulfate levels precede a decline in renal function, or *vice versa*. Elucidating this causal direction is not merely an academic exercise; it is fundamental to designing effective interventions. This will inform whether therapeutic strategies should focus on primary prevention by targeting the microbiome in at-risk individuals or on mitigating the consequences in patients with established CKD.

## A synergistic model: a multi-layered attack on the host

The impact of *F. nucleatum* on CRC pathogenesis cannot be explained by a single pathway; rather, its effects are maximized through the synergistic action of multiple mechanisms. This section integrates the preceding analyses to present a comprehensive model of how this bacterium orchestrates a multi-layered attack on the host. *F. nucleatum* functions as a keystone pathogen that orchestrates a pro-tumorigenic polymicrobial biofilm within the tumor microenvironment.^54^ It not only employs its own arsenal of virulence factors but also interacts with other co-existing bacteria (e.g., *Peptostreptococcus stomatis* and *Parvimonas micra*) to collectively enhance these pathogenic mechanisms. For instance, the biofilm structure created by this polymicrobial community may physically shield cancer cells from immune surveillance, while metabolic cross-feeding could optimize nutrient availability, thereby fostering an environment conducive to tumor growth.^55^

Within the tumor microenvironment, *F. nucleatum* simultaneously deploys three main mechanisms. First, it directly stimulates the proliferation of genetically mutated CRC cells via the FadA/Wnt pathway. This contributes to tumor growth and an increase in cell numbers. Second, it utilizes the LPS/TLR4 pathway to release pro-inflammatory cytokines, such as IL6 and IL8, establishing a persistent inflammatory state. This inflammation not only creates an environment favorable for tumor cell survival and invasion but also forms a positive feedback loop via miR-21 to amplify the inflammatory signal. Third, through the Fap2/TIGIT pathway, it effectively evades immune system attacks. The physical characteristics of Fap2 and its binding to TIGIT neutralize the killing capacity of NK and T cells, allowing tumor cells to escape immune surveillance. In addition to these local effects, *F. nucleatum* employs indirect mechanisms. *F. nucleatum* produces indole, which is metabolized to indoxyl sulfate in the liver. This indoxyl sulfate then circulates systemically, continuously sending pro-proliferative signals to tumor cells via the AKT/β-catenin/c-Myc and AHR/c-Myc pathways. This serves as another promoter, stimulating tumor cells in distant locations, independent of local growth factors.

These multi-layered actions efficiently accelerate CRC progression. Tumor cells are directly stimulated to proliferate by FadA, while simultaneously receiving inflammatory support from LPS and protection from the immune system via Fap2. Systemically circulating indoxyl sulfate provides additional fuel for this proliferative process. This combined effect supports the view that *F. nucleatum* functions as a potent promoter, complementing the first hit of genetic mutations and dramatically accelerating tumor progression.

While other bacteria may also produce indole or urease, the potential uniqueness of *F. nucleatum* as a keystone pathogen in this axis may be attributed to its synergistic virulence. Its potent adhesin, FadA, allows for firm attachment to CRC cells, which could create a microenvironment with a locally high concentration of indole, thereby facilitating its efficient absorption and subsequent systemic effects, which non-adherent bacteria cannot achieve. This ability of its metabolic output to physically bridge the colonic epithelium may distinguish *F. nucleatum* as a particularly effective driver of this pathological cycle.

## Conclusions and future perspectives: a paradigm shift in CRC pathogenesis

**Figure 1:**
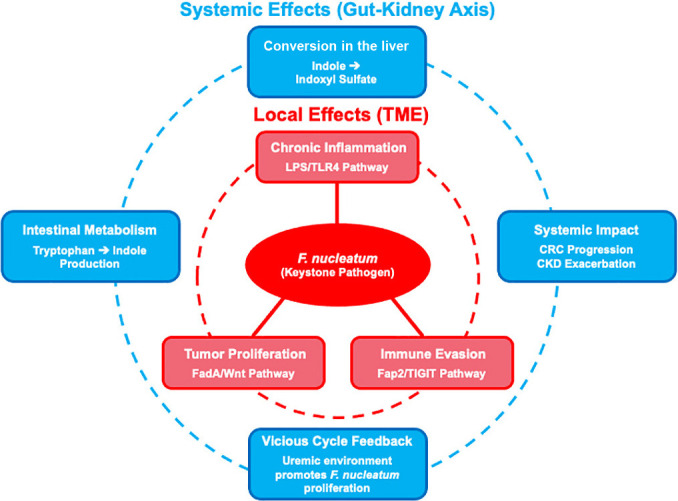
Schematic representation of the multi-layered attack by *F. nucleatum*. As a keystone pathogen, *F. nucleatum* promotes colorectal cancer (CRC) progression through local and systemic actions. Locally (inner red frame), within the tumor microenvironment (TME), it simultaneously drives three direct mechanisms: proliferation, chronic inflammation, and immune evasion. Systemically (outer blue frame), indole produced in the gut is converted in the liver to indoxyl sulfate, which then circulates throughout the body, exacerbating both CRC and chronic kidney disease (CKD). Conversely, the uremic environment induced by CKD fosters *F. nucleatum* growth, forming a self-perpetuating cycle (dashed arrow) that exacerbates disease pathology.

This review describes how *F. nucleatum* plays a critical role in the initiation and progression of CRC through multifaceted and synergistic mechanisms (Figure 1). Its pathogenic actions do not operate in isolation; rather, its direct local effects on the tumor and its indirect systemic actions via the gut–kidney axis converge to create a powerful pro-tumorigenic synergy. *F. nucleatum* contributes to a chronic pro-tumor inflammatory state via the LPS/TLR4 pathway, directly stimulates tumor cell proliferation via the FadA/Wnt pathway, and neutralizes the immune system via the Fap2/TIGIT pathway. These local effects are further amplified by a systemic metabolic pathway involving the production of indoxyl sulfate, which promotes CRC progression and exacerbates the underlying pathology of CKD, highlighting the crucial role of the gut–kidney axis.

Beyond its mechanistic insights, this gut–kidney axis model repositions serum indoxyl sulfate as a powerful integrative biomarker with significant clinical implications. In this framework, indoxyl sulfate is no longer viewed merely as a uremic toxin but as a central metabolic node linking three distinct pathologies: CRC, gut dysbiosis, and CKD. For example, elevated serum indoxyl sulfate in a patient with CRC can offer a multifaceted clinical snapshot, simultaneously suggesting 1) a highly active pro-proliferative state within the tumor, 2) a dysbiotic gut environment dominated by indole-producing bacteria, such as *F. nucleatum*, and 3) a significant concurrent risk or future progression of CKD. Viewing indoxyl sulfate through this lens transforms it into a potent biomarker for holistic risk stratification. Furthermore, urine and serum samples could be analyzed in concert to assess different stages of the disease process. In CRC patients with preserved renal function, elevated urine indoxyl sulfate levels may serve as an early and sensitive indicator of pro-tumorigenic dysbiosis driven by indole-producing bacteria, such as *F. nucleatum*, identifying a high-risk state before systemic accumulation and renal decline. Subsequently, elevated serum indoxyl sulfate levels signify the progression to a state of systemic overload and active engagement in the self-perpetuating gut–kidney cycle. Therefore, combining urine and serum indoxyl sulfate measurements could guide more comprehensive patient management, from early detection to advanced risk management, thereby addressing the interconnected pathologies of the gut, kidneys, and colorectum.

This comprehensive understanding, which integrates local synergistic mechanisms with the systemic perspective of the gut–kidney axis, provides a more complete picture of CRC pathogenesis. It indicates that a holistic view of the polymicrobial ecosystem and its interactions with the host’s genetic background is essential for a paradigm shift in the field. To achieve this, several critical questions remain unanswered. Future research must elucidate the nature of polymicrobial interactions in the tumor microenvironment, moving beyond simple co-occurrence to functional analyses using advanced multi-omics approaches. It is crucial to quantify the specific contribution of *F. nucleatum* to the systemic pool of the uremic toxin, indoxyl sulfate. As proposed, integrating metabolomics, metagenomics, and gnotobiotic animal models will be key to clarifying whether its role as an indole producer is sufficient to drive the self-perpetuating cycle of the gut–kidney axis. Answering this question is fundamental to validating a core component of our synergistic model and will be a critical step in determining whether targeting *F. nucleatum*’s metabolic activity could be a viable therapeutic strategy for both CRC and CKD. Specifically, the combination of spatial transcriptomics and 16S rRNA-targeted fluorescence *in situ* hybridization enables the visualization of direct interactions between *F. nucleatum* and specific cell types within the tumor microenvironment, such as tumor and immune cells, providing unprecedented insight into the physical dynamics of its pathogenic effects.^56^ Similarly, addressing the challenge of *F. nucleatum* strain diversity by identifying highly conserved immunogenic epitopes is crucial for developing effective vaccines.

By functioning as a powerful promoter, *F. nucleatum* complements the first hit of genetic mutations and accelerates tumor progression. This understanding provides a foundation for developing innovative diagnostics and therapies that target this key microbe to improve the prognosis for patients with CRC. The clinical translation of research findings is rapidly advancing. Strategies range from direct elimination of *F. nucleatum* to modulating its intratumoral activity to enhancing other cancer treatments, such as sonodynamic therapy.^57^ The most prominent approaches—antibiotics, phage therapy, and vaccines—each present unique benefits and challenges.

• Antibiotics: The use of antibiotics, such as metronidazole, is currently being tested in clinical trials (e.g., NCT06569368) as a sensitizer to enhance the efficacy of standard neoadjuvant therapy.^58^ This approach is promising for immediate clinical applications. However, significant concerns remain, including the risk of promoting antibiotic resistance and the potential for off-target effects on the broader beneficial microbiome, which could inadvertently exacerbate gut dysbiosis.^59^
• Phage therapy: Phage therapy offers the significant advantage of targeted specificity.^60^ Bacteriophages can be selected to eliminate *F. nucleatum* with surgical precision, thereby minimizing disruption to the commensal gut bacteria that are essential for host health. The primary hurdle is the isolation and characterization of a broad-spectrum phage cocktail capable of targeting the diverse pathogenic strains of *F. nucleatum* in patients.^61^
• Vaccines: A therapeutic vaccine targeting key adhesins, such as FadA and Fap2, represents another highly specific and promising long-term strategy.^62^ The central challenge lies in overcoming the significant strain diversity of *F. nucleatum*.^19^ The success of this approach depends on the identification of highly conserved immunogenic epitopes shared across all pathogenic clades, ensuring broad protection and preventing immune escape.

Ultimately, future treatment will likely involve a personalized medicine approach, using multi-marker diagnostic panels to identify patients who would benefit most from these targeted therapies. The clinical implications and potential therapeutic strategies are summarized in Table 4.

## Disclosure Statement

The author (H.S.) declares no conflict of interest.

## Funding Sources

This work was supported by a Sustainable Development Goals Research Project of Shimane University.

## Ethical Approval

Ethical review was not required for this study because it did not involve human participants or identifiable human data.

## Figures and Tables

**Table 1:** Stage-specific microbial and metabolic signatures in CRC development

Stage	Key bacteria elevated	Key metabolites altered
Multiple polypoid adenomas	*Atopobium parvulum*, *Actinomyces odontolyticus*	↑ Deoxycholate
Intramucosal carcinoma (S0)	*F. nucleatum*, *Solobacterium moorei*, *A. parvulum*	↑ Branched-chain amino acids, ↑ Phenylalanine, ↑ Conjugated bile acids
Advanced CRC (SI/II, SIII/IV)	*F. nucleatum*, *Peptostreptococcus stomatis*, *Parvimonas micra*	↑ Isovalerate

*Note*: Based on Yachida et al., 2019.

**Table 2:** Molecular mechanisms by which *F. nucleatum* affects CRC pathogenesis and clinical implications

Pathogenic pathway	Key virulence factor/metabolite	Host receptor/target	Core cellular effect	Tumor microenvironment impact (pro-carcinogenic action)	Potential therapeutics
LPS/TLR4 pathway	Lipopolysaccharide	TLR4	NFκB activation → Pro-inflammatory cytokine production (e.g. IL6, IL8)	Creation of a chronic inflammatory tumor microenvironment that promotes cell proliferation, survival, and invasion.	TLR4 antagonists, anti-cytokine therapies
Fap2/TIGIT pathway	Fap2 Adhesin	TIGIT on NK cells and T cells	Suppression of NK/T cell cytotoxic activity	Immune evasion and establishment of an immune-privileged niche for tumor growth.	Anti-TIGIT antibodies (immune checkpoint inhibitors), Fap2 inhibitors
FadA/Wnt pathway	FadA Adhesin	E-cadherin	β-catenin nuclear translocation → Wnt signaling activation	Direct promotion of tumor cell proliferation via oncogenes (c-Myc, Cyclin D1); acts as a “second hit.”	FadA inhibitors, Wnt/β-catenin pathway inhibitors
Indoxyl sulfate pathway	Indole (*F. nucleatum*-derived) → indoxyl sulfate (liver-metabolized)	AHR and AKT	Activation of AHR/c-Myc & AKT/β-catenin/c-Myc pathways	Systemic pro-proliferative signaling independent of the local tumor microenvironment; enhances EGFR sensitivity.	Indoxyl sulfate reduction strategies (e.g., oral adsorbents such as AST-120), tryptophan metabolism modulation

**Table 3:** Pathophysiology of the vicious cycle in the gut–kidney axis

Stage	Driver	Mechanism and consequence
**Stage 1:** Impact of chronic kidney disease on the gut	Uremic toxin accumulation	**Mechanism:** Accumulation of urea and other uremic toxins in the gut lumen; changes in gut pH and motility. **Consequence:** Gut microbiota dysbiosis; increased intestinal permeability (“leaky gut”); selective pressure favoring proliferation of urease- and indole-producing bacteria (e.g., *F. nucleatum*).
**Stage 2:** Impact of *F. nucleatum* and metabolites on the host	Bacterial metabolites and systemic inflammation	**Mechanism:** Production of uremic toxins (e.g., indoxyl sulfate, LPS); indoxyl sulfate-induced intestinal inflammation and barrier disruption; pro-inflammatory signaling to the kidney via systemic circulation. **Consequence:** Aggravation of renal fibrosis; amplification of systemic inflammation; pro-proliferative signaling to CRC cells.
**Stage 3:** Continuation of the vicious cycle	Compromised gut barrier	**Mechanism:** indoxyl sulfate-induced intestinal barrier dysfunction facilitates the translocation of *F. nucleatum* into systemic circulation. **Consequence:** Enhanced colonization of the CRC tumor microenvironment; perpetuation of a systemic pro-inflammatory and pro-proliferative state.

**Table 4:** Clinical implications and therapeutic strategies

Category	Approach	Details and challenges
Diagnostic biomarkers (diagnosis and prognosis)	*F. nucleatum* and metabolite detection	Measuring the abundance of *F. nucleatum* (esp. Fna C2 subtype [*F. nucleatum* subsp. *animalis* C2 clade]) in tumor/fecal samples. Measuring blood concentration of microbiota-derived metabolites, such as indoxyl sulfate.
Therapeutic strategies	Elimination of bacteria	**Approach:** Specific antibiotics (e.g., metronidazole) to reduce *F. nucleatum* burden and inhibit tumor growth. **Challenges:** Risk of antibiotic resistance and disrupting beneficial gut microbiota. Efficacy in humans is under investigation.
Therapeutic strategies	Adhesion/immune evasion prevention	**Approach:** Development of small-molecule inhibitors or nanomedicines to neutralize FadA and Fap2. **Challenges:** Strategy is still in the preclinical research stage.
Therapeutic strategies	Induction of protective immunity	**Approach:** FadA is a key virulence factor promoting CRC progression; therefore, development of vaccines targeting such factors is a promising future direction.
Therapeutic strategies	Microbiota modulation	**Approach:** Phage therapy to selectively target *F. nucleatum*. **Advantages:** Highly specific, minimizing damage to beneficial bacteria. Potential to overcome antibiotic resistance.
